# Drug-induced Myopathy: Wolf at the Door?

**DOI:** 10.1210/jcemcr/luaf183

**Published:** 2025-08-22

**Authors:** Vinaya Simha

**Affiliations:** Mayo Clinic, Rochester, MN 55905, USA

**Keywords:** statin myopathy, immune necrotizing myositis, drug-induced myopathy

Confronted by both the overwhelming evidence on the benefits of statin therapy for cardiovascular risk reduction and the large number of patients in clinical practice who report statin “intolerance,” the exasperated clinician is almost akin to the villagers confronting the little shepherd in Aesop's famous fable of “The Boy Who Cried Wolf.” Multiple randomized controlled trials have established the safety and efficacy of statin therapy for primary and secondary prevention, while multiple observational studies have reported a high prevalence of statin-associated muscle symptoms (SAMS) ranging from 10% to 25% and a staggering nonadherence to treatment of almost 50% [1]. In most patients, the symptoms are mild without significant clinical sequalae, and some innovative studies have demonstrated the ability of “statin-intolerant” subjects to safely tolerate even the same statin upon retrial. Further, n-of-1 trials [2, 3] showed similar incidence of reported muscle symptoms when the same patients were randomly taking either a statin or a placebo, leading to the concept of a “statin-nocebo” effect being the primary driver of SAMS. Indeed, many guidelines suggest continuation of statin therapy in a different formulation or dose as the first step in managing patients with SAMS [4]. However, we should be vigilant about rare cases of extreme muscle toxicity, which require prompt statin discontinuation and further investigations and treatment.

In the last few months, *JCEM Case Reports* has featured at least 2 such case reports of patients who experienced statin-associated immune-mediated necrotizing myopathy (IMNM), arguably the most severe form of myotoxicity. Srinivasan et al [5] report a 64-year-old male with type 2 diabetes and coronary artery disease who presented with progressive muscle pain and weakness after having been on different statins for over 5 years. He was found to have marked elevation of muscle enzymes along with elevated titers of anti-3-hydroxy-3-methylglutaryl coenzyme A reductase (HMGCR) antibodies, suggesting the diagnosis of IMNM. Skeletal muscle biopsy confirmed the diagnosis, and he was treated with high-dose steroids and intravenous immunoglobulin (IVIG). Gomez-Perez et al [6] reported a 71-year-old female with a similar presentation after having been on atorvastatin for 5 years. In addition to glucocorticoids and IVIG, this patient was treated with rituximab, which led to the resolution of myositis.

Statin-associated IMNM is a rare complication with an estimated incidence of 2 to 3 per 100 000 statin-treated individuals. Most reported cases involve middle-aged individuals in the 5th to 7th decade who had been on statin therapy for 1 to 10 years. Along with muscle pain, there is characteristic symmetric proximal muscle weakness involving the hip or shoulder girdle, which is progressive and often debilitating. Marked elevation in creatinine kinase (CK) levels usually greater than 1000 U/L and sometimes reaching up to 100 times the upper limit of normal even after cessation of statin therapy is noted. Muscle biopsy reveals muscle fiber degeneration, regeneration, and necrosis with macrophage infiltration and helps to establish the diagnosis. However, detection of circulating anti-HMGCR antibodies has a greater than 95% sensitivity and specificity for the diagnosis of this disorder and may help avoid muscle biopsy. It would therefore be prudent to check for these antibodies in statin-treated individuals who present with severe proximal muscle weakness, sometimes accompanied by dysphagia and marked elevation in CK levels, especially when there is no improvement after holding statin therapy for a few weeks. Unlike other forms of SAMS, this is not self- limited and causes progressive weakness and debility without immune modulatory therapy. Besides high-dose glucocorticoids initially, patients often need other immune-suppressive agents such as methotrexate, azathioprine, IVIG, and rituximab for disease control. Further, these patients should never be rechallenged with another statin. Clearly the approach to patients with suspected stain-associated IMNM is very different from those who present with nonimmune toxic myopathy manifesting with myalgia and cramps that usually subside after a few weeks of statin discontinuation.

The mechanism by which statins induce IMNM remains to be determined. It is likely that in genetically predisposed individuals, statin therapy either upregulates HMGCR expression or causes conformational changes when binding to it, leading to altered antigenicity and antibody formation. However, similar patterns of IMNM have been reported in statin-naïve individuals, including children, and in association with other antibodies such as antisignal recognition particle in seronegative individuals and in association with systemic malignancy. Other drugs have also been implicated, including sodium/glucose co-transporter 2 inhibitors [7]. Interestingly, this group of drugs can also cause a nonimmune type of toxic myopathy as reported in this journal a few months ago [8].

Khan and Broce [8] reported a 47-year-old male with type 2 diabetes and heart failure who presented with constant aching of the legs and thighs 2 weeks after starting empagliflozin. CK levels were 3 to 4 times the upper limit of normal, and there was no evidence of autoimmune myositis or renal insufficiency. The patient was not on concomitant statin therapy, having previously experienced statin-induced rhabdomyolysis. His symptoms resolved within 2 weeks of stopping empagliflozin. This case illustrates the need to always consider drug-induced myopathy when patients present with generalized aches and pains after starting any new medication. The fact that this patient developed myopathy with both statin and empagliflozin suggests a possible underlying primary muscle or metabolic disorder. Many conditions such as mitochondrial disorders, myophosphorylase deficiency, and myotonic dystrophy are well recognized to increase the predisposition to severe muscle adverse effects, including rhabdomyolysis when exposed to drugs such as statins, glucocorticoids, or hydroxychloroquine. Whether the reported patient was evaluated for these underlying conditions is not detailed in the case report.

Statin and other drug-induced myopathies are commonly encountered in general practice and more so by specialists caring for patients at high cardiovascular risk, such as those with diabetes and dyslipidemia. In many patients, reassurance or a brief drug interruption before switching to a different dosing or drug formulation will suffice. However, the referenced case reports [5, 6, 8] should alert us to be vigilant of patients who may have an uncommon but severe form of myositis requiring aggressive therapy or underlying muscle or metabolic disorders predisposing to drug-induced myopathy. As in Aesop's fables, so it is in clinical practice: ignoring all cries of “wolf-wolf” may lead to disastrous consequences.
